# The TGF-β1 dynamics during radiation therapy and its correlation to symptomatic radiation pneumonitis in lung cancer patients

**DOI:** 10.1186/1748-717X-4-59

**Published:** 2009-11-27

**Authors:** Ji-Yoon Kim, Yeon-Sil Kim, Young-Kyoon Kim, Hyun-Jin Park, Seung-Joon Kim, Jin-Hyoung Kang, Young-Pil Wang, Hong-Seok Jang, Sang-Nam Lee, Sei-Chul Yoon

**Affiliations:** 1 Department of Radiation Oncology, College of Medicine, The Catholic University of Korea, Seoul, Korea; 2 Division of Pulmonology, Department of Internal Medicine, College of Medicine, The Catholic University of Korea, Seoul, Korea; 3 Department of Radiology, College of Medicine, The Catholic University of Korea, Seoul, Korea; 4 Department of Medical Oncology, College of Medicine, The Catholic University of Korea, Seoul, Korea; 5 Department of Thoracic Surgery, College of Medicine, The Catholic University of Korea, Seoul, Korea

## Abstract

**Backgroud:**

The underlying molecular and cellular mechanisms of radiation pneumonitis (RP) are very complex. Several biological factors need to be considered together with the well known dosimetric parameters for understanding the molecular events in developing RP in lung cancer patients. The aim of this study was to correlate the variations of the cytokine levels in lung cancer patients during radiation therapy (RT) with the occurrence of symptomatic RP.

**Methods:**

Thirty-four lung cancer patients who received three-dimensional conformal radiation therapy were evaluated prospectively. Serial blood samples before, at the beginning, in the middle of, at the end of RT and 2 and 4 weeks after RT were analyzed for IL-1α, IL-6, IL-10, TNF-α and TGF-β1 by performing enzyme-linked immunosorbent assay. The predictive values of dosimetric factors for RP were evaluated, too.

**Results:**

Overall, 8 patients (23.5%) had grade ≥ 2 RP. By serial measurement of cytokines level, only the TGF-β1 level showed a correlation to the symptomatic RP. None of the other cytokines, IL-1α, IL-6, IL-10 and TNF-α level was correlated with the risk of RP. The mean pretreatment TGF-β1 level did not differ between RP and non-RP groups. However, during the period of radiation treatment, the TGF-β1 level began to increase at the end of RT in the RP group and became significantly higher 4 weeks after RT (*p *= 0.007). Using an ANOVA model for repeated-measures, we found significant associations between the changes of TGF-β1 during the time course of the RT and the risk of developing RP (*p *< 0.001). Most of the dosimetric factors showed a significant association with RP.

**Conclusion:**

Our results show that the changes of TGF-β1 could be correlated with RP and the incorporation of the biological parameters into the dosimetric data could be useful for predicting symptomatic RP.

## Background

Increasing radiation dose and combining it with chemotherapy were found to improve the local control for lung cancer and thus improve the overall survival of lung cancer patients [1-4]. However, the tolerance of the surrounding normal tissues to radiation therapy limits the level of dose that can be delivered for the treatment of lung cancer.

The risk of radiation pulmonary toxicity may increase for the following patients: patients with a poor performance status or inadequate pulmonary function, patients who undergo combined chemoradiotherapy and patients receiving an increased total radiation dose and treatment volume. Dosimetric parameters such as V5, V10, V20, V30, V40, the mean lung dose (MLD) and the normal tissue complications probability (NTCP) have been reported to be related with RP by several retrospective or prospective studies [5-11]. However, the underlying molecular and cellular mechanisms of RP are very complex. Several biological factors need to be considered together with the above mentioned dosimetric parameters for understanding the molecular events in developing radiation-induced complications in normal tissue [5,8,12]. Several clinical reports have suggested a possible role of profibrogenic and proinflammatory cytokines in the modulation of radiation pulmonary injury [12-18].

We designed a prospective study of lung cancer patients who underwent radiation therapy to assess the value of the cytokine dynamics, as well as of dosimetric factors, for predicting the risk of developing symptomatic RP.

## Methods

### Patient eligibility

We prospectively included lung cancer patients who underwent radiation therapy from November 2006 to April 2007 at Seoul Saint Mary's Hospital, Seoul, Korea.

Patients eligible for this study were those who had histologically proven lung cancer, received three-dimensional conformal radiation therapy (3D-CRT) above 45 Gy and an ECOG performance status score of 0-2 with the life expectancy of more than 6 months. Written informed consents were obtained from all of the patients in accordance with the procedures of the Institutional Review Board of the Hospital.

### Clinical evaluation and treatment description

History taking and physical examination that emphasized the respiratory system were performed before, during and after RT. All of the patients underwent pretreatment chest X-ray, chest CT scan, pulmonary function tests (PFT), bone scan and if necessary, PET CT and brain MRI.

A chest CT scan including the entire lung volume was performed with the immobilization device at 3 mm scan thickness. The structures of interest, such as gross tumor volume (GTV), clinical target volume (CTV), and normal structures (esophagus, spinal cord, heart, and lung) were defined and contoured on the multiple CT images. The gross tumor volume included the tumor and clinically involved nodes. GTV was determined by chest CT and/or PET-CT. CTV included GTV with additional uniform 5 cm expansion and ipsilateral hilar lymph nodes. The uninvolved mediastinal, and supraclavicular nodal regions were not routinely included in the CTV, but high risk nodal stations near GTV nodes were included occasionally. The PTV was obtained by a 0.7~1.0 cm margin 3-D expansion from the CTV. An additional 0.5~1.0 cm margin was added to superior and inferior direction to account for respiratory motion as assessed under fluoroscopy. The beam arrangement was planned to minimize the irradiated lung volume usually using 3 to 5 coplanar oblique beams. Some patients of having bulky tumor were treated with AP/PA fields to include the CTV for the first 30~40 Gy, followed by off-cord oblique beam to the GTV usually composed of 3~5 beams. After making correction for the tissue inhomogeneity using convolution/superposition algorithm of the treatment planning system (Pinnacle, Philips Medical System, Andover, MA, U.S.A.), the mean lung dose (MLD), V5, V10, V20 and V30 (the percentages of the irradiated lung volume receiving a radiation dose exceeding 5 Gy, 10 Gy, 20 Gy and 30 Gy, respectively) were calculated from the lung Dose Volume Histogram (DVH). The lung dosimetric factors were calculated with subtraction of the GTV. The both lungs were considered either as a single paired organ or as two separate organs.

Radiation therapy was delivered with 10 MV X-ray from a LINAC (Siemens, Concord, CA, U.S.A.). The median radiation dose given was 55.6 Gy (range: 45-66 Gy) and the median fraction size was 1.8 Gy (range: 1.8-3 Gy).

### Follow up and the definition of RP

The patients visited the hospital for follow-up at 2 weeks and 1, 2, 3 and 6 months after RT. Chest X-rays were performed at every follow-up visit and chest CT was done at 1, 3 and 6 months after RT. We evaluated the symptoms and signs of RP and the radiological changes upto at least 6 months after treatment. The end point of this study was the development of ≥ grade 2 RP. Grading of the RP was recorded using the scale defined by RTOG, which is based on the severity of clinical symptoms of patients with radiographic changes. It is outlined as follows: Grade 1 pneumonitis is for asymptomatic patients or who have mild symptoms(dry coughing or dyspnea on exertion) with radiographic findings; Grade 2 pneumonitis is for patients who are moderately symptomatic(persistent coughing requiring narcotic or antitussive); Grade 3 pneumonitis is for severely symptomatic patients(severe coughing unresponsive to narcotic or antitussive or dyspnea at rest, intermittent oxygen or steroid required); Grade 4 pneumonitis is for patients with severe respiratory insufficiency who needs continuous oxygen or assisted ventilation; Grade 5 pneumonitis means death due to aggravation of pneumonia. Lung injury in the immune suppressed host is associated with diversity of etiologies: sepsis, respiratory infection, irradiation, reperfusion injury, chemotherapeutic agents and other drug reactions. Multidisciplinary Team of Lung Cancer in Seoul St. Mary's Hospital reviewed our patients suspected of having RP and excluded other confirmed causes of pneumonia that mimicked RP such as infectious pneumonia or disease progression.

### Analysis of the circulating cytokines

Peripheral blood samples were collected from the patients at six points: before treatment and at the beginning, in the middle of and at the end of RT and at 2 and 4 weeks after the completion of RT. By using meticulous handling procedure, the blood samples were collected into an EDTA-contained tube. Then within an hour, centrifugation at 3000 × g was carried out for 20 min at 4°C, the plasma supernatant collected and stored in aliquots of 500 μL at -80°C until use. The plasma IL-1α, IL-6, IL-10, TNF-α and TGF-β1 levels were determined by an ELISA kit (R&D systems inc., Minneapolis, MN, U.S.A.). To minimize the variability of each ELISA assay, we completed all of the assay with just 2 times and compared standard dose density curve of different assays to ensure reproducibility.

### Statistical analysis

Statistical analysis was performed to correlate radiation pneumonitis with various potentially predictive parameters (dosimetric variables and changes in cytokine levels). The differences in dosimetric variables and cytokine levels between RP and non-RP groups were compared by conducting the student's t-test. Analysis of variance for repeated measures was used to examine the interaction between changes of cytokine levels during time course and RP occurrence. All the calculations were performed using the SAS system (SAS Institute Inc., Cary, NC, USA). A two-sided value of < 0.05 was considered statistically significant.

## Results

### Patient Characteristics

Forty-three patients were initially included in the study, but nine patients were then excluded from analysis due to incomplete treatment (2 patients), unsatisfactory blood sampling (3 patients) and follow-up loss or a follow-up that was less than 6 months (4 patients).

For finally 34 evaluable patients, the histology was 14 squamous cell carcinomas, 6 adenocarcinomas, 11 small cell carcinomas and 3 others. Twenty-five patients were male and nine patients were female. The median patient age was 63 (age range: 42-78). The ECOG performance status was 0 or 1 for 29 patients (75%). Twenty-six patients (78%) had a smoking history. Chemotherapy was done before radiation in 21 patients (62%), and concurrent chemoradiotherapy was done in 15 patients (44%). The used regimens for concurrent chemotherapy were combinations of etoposide and cisplatin (6 patients) or docetaxel and cisplatin (4 patients). Other chemotherapeutic agents included weekly taxol (4 patients) or cisplatin (1 patient). Twelve patients (35.3%) had previous surgery for the lung cancer. The results of the pretreatment pulmonary function test for all the patients were as follows: the median FEV1 was 1.81 L (70%), the median FEV1/FVC 67% and the median DLCO 78%. The patients characteristics are summarized in Table 1.

**Table 1:** Patients characteristics (n = 34)

Characteristics		Number of patients (%)
Gender	Male	25 (74)
	Female	9 (26)
Age(yrs)	Median	63
	Range	42~78
ECOG	0	13 (38)
	1	16 (47)
	2	5 (15)
Site	Upper	22 (65)
	Lower	10 (29)
	Upper+Lower	2 (6)
Histology	Sqamous cell ca	14 (41)
	Adeno ca	6 (18)
	Small cell ca	11 (32)
	Other	3 (9)
Location	Central	26 (76)
	Peripheral	8 (24)
Previous surgery	No	22 (65)
	Yes	12 (35)
Previous chemotherapy	No	13 (38)
	Yes	21 (62)
Concurrent chemotherapy	No	19 (56)
	Yes	15 (44)
Smoking	Never	7 (21)
	Previous	13 (39
	Current	13 (39)
PFTs FEV1(L)	Median Range	1.8 0.8~4.5
FEV1/FVC(%)	Median Range	67 34~94
DLCO(%)	Median Range	78 53~133

Abbreviations: ECOG, Eastern Cooperative Oncology Group Performance Scale; PFTs, Pulmonary Function Tests

### Radiation pneumonitis

The median duration of follow up was 10 months (range: 1 - 23 months). Of the 34 patients who were included in the study, 17 patients (50%) developed any grade of RP. The severity of pneumonitis was grade 1 in 9 patients (26.5%), grade 2 in 5 patients (14.7%) and grade 3 in 2 patients (5.9%). One patients (2.9%) died of aggravation of RP. The median time to the onset of pneumonitis was 1.6 months (range: 0-3.3 months).

Among 8 patients who developed ≥2 RP, 7 patient received concurrent chemoradiation. The most frequently used regimens were combinations of etoposide and cisplatin (4 patients) or docetaxel and cisplatin (2 patients).

The patient who developed grade 5 pneumonitis was 74 year-old ex-smoker. He was newly diagnosed for squamous cell carcinoma. The stage was IIIB, so concurrent chemoradiation with weekly taxol was recommended considering his good performance status (ECOG1) and PFTs (FEV1 = 2.52L, DLCO = 84%). He was relatively well tolerable until the end of treatment. However, he had coughing, progressive dyspnea and fever with new developed consolidation corresponding to radiation field after 1.5 months completion of RT. No specific infectious cause was identified. He received intravenous steroid therapy and ventilator care, but died despite of 4 weeks of intensive care.

### Dosimetric parameters

All the dosimetric factors were analyzed for the lung both as a paired organ and as a separate organ. Most of the dosimetric factors showed an association with RP. The data are shown in Table 2. For the lung as a paired organ, V5, V10 and V20 were statistically significant factors for the occurrence of RP (*p *= 0.035, *p *= 0.049, and *p *= 0.049, respectively). The V30 and MLD were marginal significant (*p *= 0.068, *p *= 0.077). For the lung as a separate organ, the MLD, V5, V10 and V20 values were statistically significant factors for the occurrence of RP (*p *= 0.018, *p *= 0.003, *p *= 0.006 and *p *= 0.032, respectively). The V30 was marginally significant (*p *= 0.053).

**Table 2:** Dosimetric risk factors for development of RP ≥ grade 2

		Mean ± SD		
	DVH parameters	RP	Non-RP	*p *value*
Whole lung	MLD (cGy)	1188 ± 354	957 ± 382	0.077
	V5 (%)	58 ± 15	45 ± 12	0.035
	V10 (%)	43 ± 13	34 ± 13	0.049
	V20 (%)	29 ± 8	22 ± 11	0.049
	V30 (%)	22 ± 7	17 ± 9	0.068
	V40 (%)	15 ± 5	12 ± 7	0.299

Ipsilateral lung	MLD (cGy)	1986 ± 491	1512 ± 612	0.018
	V5 (%)	78 ± 13	63 ± 17	0.003
	V10 (%)	67 ± 11	52 ± 19	0.006
	V20 (%)	49 ± 11	38 ± 17	0.032
	V30 (%)	38 ± 11	29 ± 15	0.053
	V40 (%)	24 ± 9	22 ± 14	0.655

Abbreviations: RP, radiation pneumonitis; DVH, dose volume histogram; MLD, mean lung dose;

V5, the percentage of the irradiated lung volume receiving a radiation dose exceeding 5 Gy;

V10, the percentage of the irradiated lung volume receiving a radiation dose exceeding 10 Gy;

V20, the percentage of the irradiated lung volume receiving a radiation dose exceeding 20 Gy;

V30, the percentage of the irradiated lung volume receiving a radiation dose exceeding 30 Gy;

V40, the percentage of the irradiated lung volume receiving a radiation dose exceeding 40 Gy

* student's t-test

### Serum cytokine levels

#### TGF-β1

As demonstrated in Table 3, the mean pretreatment TGF-β1 level was 2.8 ± 0.8 × 10^4 ^pg/ml for the RP group and 2.3 ± 1.1 × 10^4 ^pg/ml for the non-RP group. The patients who developed pneumonitis showed a higher level of pretreatment TGF-β1, but this was not statistically significant (*p *= 0.157). During the period of radiation treatment, from the beginning of RT to the middle of RT, the RP group tended to show a decrease in the TGF-β1 level. However, the TGF-β1 level began to increase at the end of RT in the RP group and became significantly higher at 4 weeks after RT (*p *= 0.007). These elevation of TGF-β1 level after RT were same in the patients of ≥ grade 2 RP (p = 0.062). However, TGF-β1 level of patients who did not develop RP began to decrease relative to their pretreatment level after middle of RT.

**Table 3:** Changes of mean TGF-β1 level during the course of radiation therapy

		Mean ± SD (× 10^4 ^pg/ml)		
	Time	RP	Non-RP	*p *value*
RP1	Before RT	2.8 ± 0.8	2.3 ± 1.1	0.157
(n = 17)	Beginning of RT	2.4 ± 0.7	2.5 ± 1.1	0.738
	Middle of RT	2.0 ± 0.6	2.4 ± 1.2	0.19
	End of RT	2.1 ± 0.7	2.2 ± 0.9	0.879
	2 wks after RT	2.1 ± 0.4	2.0 ± 0.8	0.806
	4 wks after RT	2.8 ± 1.1	1.7 ± 0.5	0.007

RP2	Before RT	2.5 ± 1.0	2.6 ± 0.9	0.844
(n = 8)	Beginning of RT	1.9 ± 0.6	2.6 ± 0.9	0.079
	Middle of RT	1.8 ± 0.7	2.3 ± 0.9	0.168
	End of RT	1.9 ± 0.6	2.2 ± 0.8	0.352
	2 wks after RT	2.1 ± 0.7	2.0 ± 0.9	0.787
	4 wks after RT	3.3 ± 1.7	2.0 ± 0.9	0.062

Abbreviations: RT, radiation therapy; wks, weeks; RP1, development of any grade of radiation pneumonitis; RP2, development of grade ≥ 2 radiation pneumonitis;

* student's t-test

**Table 4:** Results of repeated measures ANOVA about the changes of mean TGF-β1 level during the course of radiation therapy

	Mean ± SD (× 10^4 ^pg/ml)						Repeated measures ANOVA		
	Before RT	Beginning of RT	Middle of RT	End of RT	2 wks after RT	4 wks after RT	Source	F	*p *value
RP1 (n = 17)	2.8 ± 0.8	2.4 ± 0.7	2.0 ± 0.6	2.1 ± 0.7	2.1 ± 0.4	2.8 ± 1.1	RP1	0.28	0.598
Non-RP (n = 17)	2.3 ± 1.1	2.5 ± 1.1	2.4 ± 1.2	2.2 ± 0.9	2.0 ± 0.8	1.7 ± 0.5	time	2.36	0.043
							time*RP1	4.79	0.001

RP2 (n = 8)	2.5 ± 1.0	1.9 ± 0.6	1.8 ± 0.7	1.9 ± 0.6	2.1 ± 0.7	3.3 ± 1.7	RP2	0.05	0.828
Non-RP	2.6 ± 0.9	2.6 ± 0.9	2.3 ± 0.9	2.2 ± 0.8	2.0 ± 0.9	2.0 ± 0.9	time	3.24	0.008
(n = 26)							time*RP2	6.27	0.0001

Abbreviations: RT, radiation therapy; wks, weeks; RP1, development of any grade of radiation pneumonitis; RP2, development of grade ≥ 2 radiation pneumonitis

The pretreatment TGF-β1 level of the patient who died of RP was relatively high (2.9 × 10^4 ^pg/ml). The TGF-β1 level decreased during RT as same as other RP patients. His TGF-β1 level at 4 weeks after RT was markedly increased upto 4.2 × 10^4 ^pg/ml.

**Figure 1:**
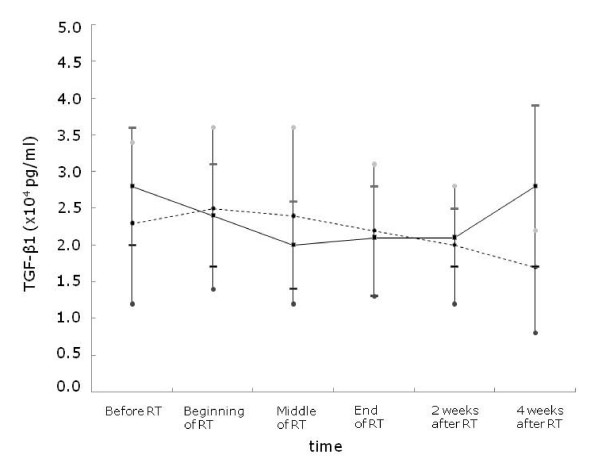
**The pattern of the changes of the mean TGF-β1 level in the RP group and the non-RP group**. The TGF-β1 level began to increase at the end of RT in the RP group and it became significantly higher at 4 weeks after RT (*p *= 0.007). However TGF-β1 level of patients who did not develop RP began to decrease relative to their pretreatment level after middle of RT. The solid line shows the mean level of TGF-β1 in the RP group and the dashed line shows the mean level of TGF-β1 in the non-RP group. The data are presented as mean ± standard error of the mean.

We performed an ANOVA model for repeated-measures for analysis of chronological change in TGF-β1 level and found that there were significant associations between the changes of TGF-β1 level during the time course of radiation and the risk of developing RP (*p *< 0.001 for development of any grade of RP, *p *< 0.0001 for development of grade ≥2 RP). These chronological changes in the serial TGF-β1 levels are demonstrated in Table 4, Fig 1.

#### IL-6

The pretreatment IL-6 level was higher in the non-RP group compared to the RP group (49.1 ± 123.3 pg/ml vs 16.7 ± 23.9 pg/ml, respectively, *p *= 0.387). However, the IL-6 level between each patient showed wide variation compared to the TGF-β1 level, which showed a relatively stable range of variation. The IL-6 level was low before and during RT, but it began to increase after RT in both groups. The changes of the IL-6 during the time course were similar whether patients developed RP or not. Our data showed a wide range of variation in the circulatory IL-6 levels, but relatively little changes with the development of RP.

#### Other cytokines

The IL-1α, IL-10 and TNF-α level were stable during the whole course of RT and they were not correlated with the risk of RP (data not shown).

## Discussion

Being aware of the risk of radiation pulmonary injury is an important aspect of patient management in the era of combination of chemoradiation for treating lung cancer. The recent studies on the new effective chemotherapeutic agents or hyperfractionated RT have reported that the incidence of grade ≥ 3 RP in patients who underwent concurrent chemoradiotherapy is between 21-23%[6,7]. Along with combined chemotherapy, other patient-specific or treatment-specific factors have been identified as predictors of developing RP. However, currently, there are no generally accepted methods available to accurately predict an individual patient's risk of developing RT-induced pulmonary morbidity. The purpose of this study was to assess the values of the cytokine dynamics and dosimetric factors to predict the risk of developing symptomatic RP.

The dose of irradiation administered to patients is distributed in a 3-D volume on the DVH. According to a literature review, dosimetric factors such as V5, V10, V20, V30, V_eff _(effective volume) and MLD have statistically significant correlation to symptomatic radiation pneumonitis [9-11]. Also in the current study, most of the dosimetric factors showed an association with RP. Because the lung function may not be uniform across all regions of the lung, it is unlikely that such a simplistic dose-volume relationship exists. However, these parameters are easy to calculate and useful in the clinical setting.

Although dosimetric factors are important, these factors don't take into consideration the molecular biological events that may be responsible for radiation-induced responses of normal tissue. Molecular events have been shown to occur much earlier than the clinically apparent radiation responses. Exposure to ionizing radiation rapidly triggers a cascade of genetic and molecular events, which is an active process involving the production of a number of inflammatory and fibrogenic cytokines by various cells in lung, for example, macrophages, epithelial cells, endothelial cells, pneumocytes and fibroblasts. These molecular processes are perpetuated beyond the time point at which the acute insult has been removed. Several recent studies have shown that cytokines (IL-1, IL-6, IL-8, IL-10, TNF-α, platelet-derived growth factor and TGF-β), surfactant apoproteins and cell adhesion molecules (ICAM-1, E-selectin) have important roles in RT-induced pulmonary injury [8,9,12-18]. A study of the dynamics of the serum cytokines during the early course of the treatment would be useful for predicting the risk of pulmonary injury and for the early intervention of pneumonitis. Because these cytokines are thought to be key mediators of lung toxicity, many of them have been examined as potential early markers for radiation pneumonitis.

Profibrogenic cytokine TGF-β1 is the most extensively investigated among the various biological markers in radiation-induced injuries. We observed that TGF-β1 decreased during RT and it began to increase at the end of RT in the patients who developed RP. There have been similar reported results for the TGF-β1 dynamics in relation to the development of pneumonitis after radiotherapy for lung cancer. Hur et al. found that TGF-β1 was decreased during RT and it was markedly increased at 2-4 weeks after the completion of RT for patients who developed symptomatic pneumonitis [14]. Kong et al. reported that TGF-β1 decreased during RT in patients with an increased pretreatment plasma TGF-β1 level, yet this didn't normalize even by the completion of treatment[19]. Other clinical studies have reported that the absolute level or the relative ratio of the TGF-β1 level showed meaningful changes during or after RT in patients who suffered RP. However, the correlated time points were different in each study and the patterns of the changes were not always same [14,18-20]. We also evaluated the TGF-β1 ratios (the ratios of a value from a particular time-point divided by the pre-RT value) presented in table 5. In the patients who experienced grade ≥2 RP, the TGF-β1 ratios tended to show a decrease than that of the non-RP group, from the beginning of RT to the end of RT. However, the TGF-β1 level began to increase at the 2 weeks after RT in the RP group and became more higher at 4 weeks after RT (1.6 ± 1.0 vs 0.8 ± 0.3, *p *= 0.081). The time point predictive of RP is different from the data of Zhao et al which was 4 weeks during 6 weeks' course of RT [20]. A recent report from Zhao et al suggests the combination of TGF-β1 and MLD may help stratify the patients for their risk of RP to improve the predictive power [21]. Their data show the incidence of RP was 4.3% in patients with a TGF-β1 ratio ≤ 1 and MLD ≤ 20 Gy, and 66.7% in those with a TGF-β1 ratio ≥ 1 and MLD ≥ 20 Gy.

**Table 5:** Changes of mean TGF-β1 ratio during the course of radiation therapy

		Mean of Ratio ± SD		
	Time	RP	Non-RP	p value*
RP2	Before RT			
(n = 8)	Beginning of RT	0.8 ± 0.2	1.0 ± 0.2	0.019
	Middle of RT	0.8 ± 0.4	0.9 ± 0.4	0.238
	End of RT	0.8 ± 0.2	0.9 ± 0.3	0.346
	2 wks after RT	1.0 ± 0.6	0.9 ± 0.3	0.576
	4 wks after RT	1.6 ± 1.0	0.8 ± 0.3	0.081

Abbreviations: RT, radiation therapy; wks, weeks; RP2, development of grade ≥ 2 radiation pneumonitis

* student's t-test

The TGF-β1 levels in bronchoalveolar lavage(BAL) fluid from the irradiated area increased continuously during and after RT compared to the pretreatment levels in the RP group[17]. On the other hand, in several studies the pattern of the changes of the TGF-β1 level was not distinct between the RP and non-RP groups [12,15]. The reasons for such conflicting results may be explained by the fact that numerous factors can falsely increase TGF-β1 levels and confound their predictive value for RP occurrence. First, the tumor stroma may be responsible for the production of TGF-β1 in lung cancer patients. The mean TGF-β1 level in lung cancer patients was higher than that in the normal controls (*p *< 0.001). According to a pathology slide review, the degree of fibrosis that is present in tumor is also significantly correlated with an elevated plasma TGF-β1 level (*p *= 0.03). The dynamics of the plasma TGF-β1 have been suggested to be a marker of RT-induced normal tissue injury as well as a marker of tumor response. After radiotherapy, the patients who were alive with disease had significantly higher TGF-β1 levels than those who were alive with no evidence of disease (*p *= 0.02) [19] Second, careful handling of the sample is also important. Variations of the centrifugation conditions and platelet contamination could artificially elevate plasma TGF-β1 level[19,22]

IL-6 is a pleiotropic inflammatory cytokine that is important in regulating immunologic and inflammatory responses. IL-6 levels before, during and after thoracic RT were significantly higher in those patients who developed pneumonitis in several reports [13,23]. Especially Arpin et al reported covariation of proinflammatory cytokine (IL-6) and anti-inflammatory cytokine (IL-10) levels during the first 2 week of RT were independent predictive evidence of RP. However, other studies have failed to find a relationship between IL-6 and the radiation-induced pulmonary symptoms, like what our results have shown [15,17]. In this study, the IL-6 level was low before and during RT, but it began to increase after RT. These findings are similar those of Chen et al [24]. However, the changes of the IL-6 level were similar whether patients developed RP or not. This suggests that the IL-6 produced by the lung is not a major determinant of the circulating IL-6 levels. The mean IL-6 concentrations were significantly higher in the lung cancer patients than in the normal controls, and the patients with metastatic tumor had higher IL-6 levels than those patients with undisseminated disease, suggesting that neoplastic cells may produce IL-6 [25]. IL-6 is an acute phase inflammatory cytokine, and this would suggest that the measurement of circulating IL-6 can reflect the inflammatory state of the lung. The IL-6 levels frequently increase in patients suffering with several pulmonary diseases, including infectious pneumonia, interstitial pneumonia and chronic obstructive pulmonary disease[17].

There are other potential biological predictors of RP, but none has been conclusively demonstrated to identify the patients who are at a high risk of radiation-induced pulmonary toxicity [9,12,15,16]. Host-associated diseases, the proportion of gross disease at the time of irradiation and pre-RT treatment such as chemotherapy are all associated with local cytokine production [5,15]. The addition of radiation on a background of subclinical damage may augment a cytokine cascade and increase the severity of acute and late side effects.

Although various cytokines are important in the pathogenesis of radiation-induced pulmonary injury, our results show that the changes of TGF-β1 could be an independent predictor of developing RP and well correlated with the time course of radiation therapy. However, the role of cytokine markers in the cytokine cascades that promote pulmonary injury deserves further investigation. Also, treatment strategies designed to block this pathologic process may need to be continued well beyond the completion of RT.

## Conclusion

Although the current study had a limited number of patients, we demonstrated that, in the patients who developed RP, the TGF-β1 level decreased during RT and began to increase at the end of RT. And the TGF-β1 level at 4 weeks after RT was significantly higher than the TGF-β1 level of the patients who didn't develop RP. TGF-β1 may contribute to the process leading to a radiation injury in human lung tissue. While the change of TGF-β1 level did not take place early in time course of RT with having a predictive value in our study, the incorporation of the biological parameters into the dosimetric data that have been developed to predict radiation-induced lung injury may improve the predictive accuracy. Further research must continue to identify biomarkers that will one day allow us to tailor our therapies in response to the highly accurate predictions of risk for the development of radiation pneumonitis.

## Competing interests

The authors declare that they have no competing interests.

## Authors' contributions

JYK performed the collection of blood samples, acquisition of clinical data and drafted the manuscript. YSK designed and coordinated the study, checked statistical results, read and edited the manuscript. YKK coordinated and performed laboratory work. HJP interpreted radiological findings. SJK, JHK, YPW, SCY, SNL and HSJ performed evaluation of patients and read the manuscript. All the authors read and approved the final manuscript.
